# An Excellent Functional Recovery Following Grade IV Subarachnoid Hemorrhage From a Cerebral Aneurysm Rebleed With Ultra-Early Surgical Intervention: A Case Report

**DOI:** 10.7759/cureus.47197

**Published:** 2023-10-17

**Authors:** Rohan S Pendse, Luis F Castro, Sarosh Din, Yesenia Barrios, Benedicto C Baronia

**Affiliations:** 1 Department of Neurological Surgery, Texas Tech University Health Sciences Center, Lubbock, USA; 2 Department of Neurology, Texas Tech University Health Sciences Center, Lubbock, USA; 3 School of Osteopathic Medicine, William Carey College of Osteopathic Medicine, Hattiesburg, USA; 4 Department of Emergency Medicine, Texas Tech University Health Sciences Center, Lubbock, USA

**Keywords:** endovascular coiling, hemorrhagic stroke, ultra-early surgical clipping, aneurysmal rupture, aneurysm

## Abstract

Aneurysms are focal abnormal dilations of the arterial wall occurring frequently at branching points along the arteries of the base of the brain. Aneurysmal rupture is one of the possible aneurysm complications and can cause aneurysmal subarachnoid hemorrhages (aSAH). Treatment of aSAH consists of pharmacologic, surgical, or endovascular approaches. The ultra-early intervention of ruptured aSAH occurs within the first 24 hours after ruptured aSAH. This case is about a 49-year-old obese male with multiple comorbidities who suffered from a grade IV subarachnoid hemorrhage and underwent an ultra-early surgical clipping approximately four hours after admission to the emergency center. The patient had excellent functional recovery at a six-month follow-up. Ultra-early surgical intervention for high-grade aSAH with rebleeding could improve outcomes.

## Introduction

This article was previously presented as a meeting poster at the 2023 TTUHSC Annual Student Research Week on February 28, 2023.

Aneurysms are focal abnormal dilations of the wall of an artery and are estimated to occur in about 5% of the population [1]. They commonly occur at branching points along the arteries of the base of the brain, with the most common site being the anterior communicating (ACom) artery [2]. Ruptured aneurysms cause aSAH, accounting for approximately 5% of strokes [3]. aSAH mortality estimates range between 32% and 67%, with about 25% of fatalities occurring in the first 24 hours of receiving medical treatment [4]. Rebleeding is a poor prognostic marker for functional recovery and carries an estimated mortality of 20% to 60% [5]. A large observational prospective cohort study at a university center found that the cumulative risk of rebleeding is highest in the first 24 hours after the initial event. This study also found that patients presenting with a modified Fisher grade of 3 to 4 were at significant risk of rebleeding within the first 24 hours [6].

Treatment modalities for aSAH involve pharmacologic, surgical, and endovascular approaches. Historically, surgical intervention was done within seven days of the event, but researchers showed that patients experienced better outcomes if it was done within the first 72 hours [7,8]. Furthermore, evidence shows improved outcomes in patients who underwent ultra-early surgical intervention within 24 hours of ictus [9-11]. This case report concerns a 49-year-old male who underwent ultra-early surgical intervention after an aSAH rebleed and had excellent functional recovery during his six-month post-operative visit.

## Case presentation

**Figure 1:**
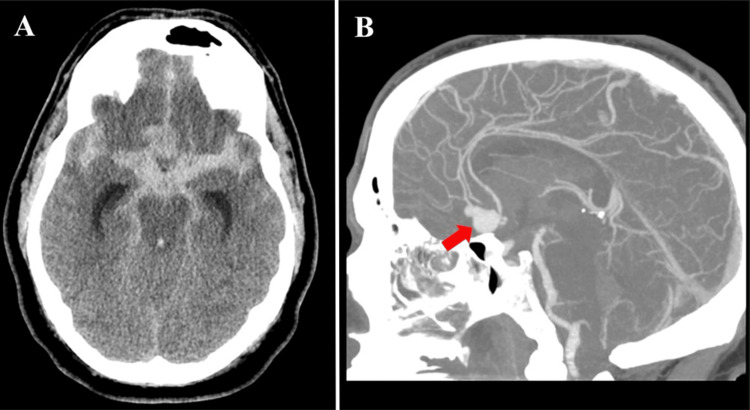
Non-contrast head CT and CT angiogram in the preoperative period showing a large anterior communicating artery aneurysm (A) Non-contrast head CT with extensive subarachnoid hemorrhage. (B) CT angiogram with a saccular aneurysm in ACom as depicted by the red arrow.

This case report is about a 49-year-old obese male with a past medical history of essential hypertension, chronic kidney disease, type 2 diabetes mellitus, and obstructive sleep apnea who presented to the emergency center after a syncopal episode during physical activity. The emergency technicians on the field put the patient on a laryngeal tube airway. On admission to the emergency center, the patient was sedated and re-intubated. Standard treatment for aSAH was instituted by initiating nimodipine for vasospasm prophylaxis, levetiracetam for seizure prophylaxis, and relative cooling measures were started. On physical examination, the patient was sedated with a Glasgow Coma Scale score of 3T. The patient was tachycardic and unresponsive to painful stimuli, and his pupils were equally round and reactive to light. Computed tomography (CT) of the head showed a large SAH (Figure 1A), and CT angiogram (Figure 1B) revealed a large saccular aneurysm in the ACom with active bleeding. The patient was taken to the operative room for an emergent ventriculostomy. During the procedure, his ventricular drain revealed frank blood, and his pupils were fixed and dilated. A decision was made to convert the procedure into a bilateral frontal decompressive craniotomy for aneurysm clipping and reconstruction. The surgery occurred approximately 3 hours and 58 minutes after presenting to the emergency center. During the procedure, mannitol was given to reduce intracranial swelling, and the aneurysm was successfully clipped. The postoperative course was uncomplicated. The patient struggled with generalized weakness, likely secondary to a prolonged hospitalization course and mild speech impairment. However, the absence of major focal neurological deficits suggested good recovery. The patient underwent a cranioplasty and was discharged three months later to a neurorehabilitation facility with outpatient follow-up. Approximately six months later, he was seen in the outpatient clinic for postoperative follow-up and was found to be doing excellent clinically.

## Discussion

The patient discussed in this case came in with a grade IV subarachnoid bleed. Given his mental status in the emergency center, he did not fit the classic presentation for aSAH. As discussed in the introduction, his aneurysm was found in the ACom artery, the most common location for saccular aneurysms. Typically, severe aneurysmal bleeds are managed with ventriculostomy to reduce intracranial pressure and the risk of herniation. However, given the acute decline caused by rebleeding of the aneurysm, the procedure was converted to an open decompressive bilateral frontal craniectomy with surgical clipping. Despite the severity of the initial presentation and rebleeding, which carries a significant mortality rate, the patient was doing excellent clinically at his six-month follow-up visit.

Functional outcome after aneurysmal SAH depends on several factors, including the severity of initial hemorrhage, rebleeding, perioperative medical management, timing, and technical success for aneurysm exclusion from the cerebral circulation [12]. Factors that can indicate poor prognosis at onset include posterior circulation rupture, older age of the patient, large aneurysm, intracerebral hematoma (ICH), intraventricular hemorrhage (IVH), extensive hemorrhage on CT scan, and past medical history of myocardial infarction, hypertension, and liver disease [13]. A comprehensive systematic review found that approximately one-third of aSAH patients achieve a favorable functional outcome [14]. Another retrospective study further demonstrated that factors influencing favorable functional outcomes included aneurysm treatment with coil embolization, better modified Fisher grade, absence of ICH, IVH, or hydrocephalus, good clinical grade, blood transfusion during hospitalization, and absence of radiological infarction [15]. However, few studies have examined the timing of surgical or endovascular intervention and functional outcomes of poor-grade aSAH. Potential factors that could have contributed to excellent recovery after aSAH include young age, intact pupillary light reflex, absence of intracerebral hemorrhage, delayed cerebral ischemia, symptomatic vasospasm, in-hospital seizures, or occurrence of hydrocephalus [15,16]. Newer studies seem to contradict the results of another extensive systematic review and meta-analysis that found no difference in clinical outcomes for ultra-early intervention compared with delayed treatment [17].

## Conclusions

Variables indicative of poor predictive outcome include a history of hypertension and diabetes, severity on presentation, extensive bleeding on CT scan, intraventricular hemorrhage, and aneurysm rebleeding. Potential factors that could have contributed to excellent recovery after aSAH include young age, intact pupillary light reflex, absence of intracerebral hemorrhage, delayed cerebral ischemia, symptomatic vasospasm, in-hospital seizures, or occurrence of hydrocephalus. An additional potential protective factor and the focus of the current case report is the ultra-early surgical intervention this patient received. It is possible that the patient discussed in this case report benefitted greatly from his relatively young age and absence of severe medical comorbidities coupled with the ultra-early surgical intervention. More research with randomized clinical trials could better elucidate the potential benefit of ultra-early surgical and endovascular intervention following aSAH compared to delayed treatment.
